# The Role of Thyroid Elastography in Children with Type 1 Diabetes Mellitus or Celiac Disease Who Have Negative Thyroid Autoantibodies

**DOI:** 10.3390/jcm15082840

**Published:** 2026-04-09

**Authors:** Arzu Gülseren, Serkan Bilge Koca, Tuğba Uylar Seber, Esra Eren, Buket Daldaban Sarıca

**Affiliations:** 1 Department of Pediatric Gastroenterology and Hepatology, Kayseri City Education and Research Hospital, Kayseri 38080, Turkey; esraeah@hotmail.com (E.E.); buketdaldaban@gmail.com (B.D.S.); 2 Department of Pediatrics, Division of Pediatric Endocrinology, Kayseri City Education and Research Hospital, Kayseri 38080, Turkey; kocaserkanbilge@yahoo.com.tr; 3 Department of Radiology, Kayseri City Education and Research Hospital, Kayseri 38080, Turkey; tuylar@gmail.com

**Keywords:** thyroid elastography, celiac disease, type 1 diabetes mellitus, antibody, anti-thyroperoxidase, anti-thyroglobulin, thyroid, autoimmune

## Abstract

**Background/Objectives**: Autoimmune thyroiditis affects physical and cognitive development in children. Therefore, early detection can prevent symptoms that could lead to lifelong changes. Autoimmune thyroiditis can frequently accompany type 1 diabetes (T1DM) and celiac disease (CD). The goal in this study is to evaluate its usability as a screening method by assessing thyroid elasticity in children with negative thyroid autoantibodies and T1DM or CD. **Methods**: This cross-sectional, case–control, single-center study was conducted with children who had applied to the Pediatrics outpatient clinic of Kayseri City Education and Research Hospital (Turkey). The study included three groups of cases (T1DM, CD and control). The value of the shear wave elastography (SWE) color map was recorded in kPa. Comparisons between two independent groups were conducted using either Student’s *t*-test or the Mann–Whitney U-test, while categorical variables were analyzed with the Chi-square test. A correlation analysis was conducted to evaluate the relationship between the variables. **Results**: The study cohort comprised 185 children, of whom 71 had T1DM, 54 had CD, and 60 constituted the healthy control group. The participants ranged in age from 4 to 17.9 years, with a mean age of 11.4 ± 3.8 years. The gender distribution did not differ significantly between the groups. Anti-thyroid peroxidase (TPOAb) levels did not differ significantly between the groups (*p* = 0.894). Thyroid volume or standard deviation score did not differ significantly between the groups. Corresponding SWE values in the T1DM, CD and control groups were 7.7 (6.0–9.3), 5.9 (5.2–7.9) versus 7.1 (6.0–9.6), respectively (*p* = 0.002). Correlations were significantly associated between SWE scores and anti-thyroglobulin (TgAb), thyroid volume, mean hemoglobin A1c (HbA1c), and time elapsed from a diagnosis of CD. **Conclusions**: The SWE scores were observed to be higher in children with T1DM compared to those with CD.

## 1. Introduction

The most prevalent endocrine disorder is autoimmune thyroiditis [1]. Approximately 1–2% of children are affected by Hashimoto’s thyroiditis (HT), a chronic autoimmune thyroiditis [2]. Given its effects on physical and cognitive development in children, the importance of early diagnosis of thyroiditis is clear. The coexistence of autoimmune thyroiditis is frequently observed in the presence of other autoimmune diseases, such as type 1 diabetes mellitus (T1DM) and celiac disease (CD) [3]. Consequently, in the context of T1DM and CD, patients are subjected to regular screening for HT, with biochemical assessments of thyroid function and serological tests employed as diagnostic tools. However, the majority of cases of autoimmune thyroiditis are asymptomatic and euthyroid, particularly at the outset.

It has been demonstrated that proinflammatory cytokines and chemokines induce thyroid cell apoptosis as a consequence of the involvement of cellular immunity in autoimmune thyroid diseases. Consequently, it has been shown that chronic autoimmune inflammation can occur in the absence of autoantibodies [2]. For pediatric patients who have not yet established a biochemical diagnosis but are at risk, may be use of imaging as a predictive tool prior to diagnosis. Histological studies have demonstrated that lymphocyte infiltration is a primary mechanism underlying the development of fibrosis in autoimmune thyroiditis [4]. The reduction in elasticity resulting from fibrosis can be quantified through the use of shear wave elastography. Clinical studies have found that thyroid elasticity is impaired in children with autoimmune thyroid diseases [5]. Thyroid stiffness did not differ significantly with age. Mean thyroid shear wave elastography (SWE) values showed no difference when comparing the first six decades in 10-year increments [6]. Therefore, it was concluded that age could be disregarded when forming groups in future studies [6]. There is a paucity of data regarding thyroid elasticity in pediatric patients with T1DM or CD who are negative for thyroid autoantibodies. Emerging evidence suggests that thyroid inflammation and fibrosis may develop before the appearance of detectable autoantibodies, indicating that seronegative patients may still have subclinical thyroid involvement. It could help clinicians better stratify patients for follow-up and optimize monitoring strategies. Additionally, as a non-invasive method, SWE may complement routine screening without increasing patient burden.

Shear wave elastography (SWE) has emerged as a non-invasive imaging modality that quantitatively assesses tissue stiffness and provides additional diagnostic information beyond conventional ultrasound in thyroid diseases. There are studies showing that sonographic elastography evaluation of thyroid nodules in the pediatric population can be used to differentiate between benign and malignant nodules [7,8]. Recent studies have shown that SWE can detect increased thyroid stiffness associated with fibrosis and autoimmune inflammation, supporting its role in the evaluation of chronic autoimmune thyroiditis. Evidence indicates that SWE may serve as a valuable tool in diagnosing autoimmune thyroiditis in the pediatric population [9,10,11]. In Türkiye, CD is frequently associated with autoimmune thyroiditis (8%) [12].

Therefore, this study aimed to evaluate whether thyroid stiffness measured by SWE could detect early thyroid involvement in children with T1DM or CD who have negative thyroid autoantibodies. We hypothesized that children with T1DM or CD, despite having negative thyroid autoantibodies, would exhibit increased thyroid stiffness compared to healthy controls.

## 2. Materials and Methods

This cross-sectional, case–control, single-center study was conducted with children who had applied to the Pediatrics outpatient clinic of Kayseri City Education and Research Hospital. The study was approved by the local ethics committee (Kayseri City Hospital Non-Interventional Clinical Research Ethics Committee, decision date: 4 April 2023, decision number: 821). Written informed consent form was obtained from parents of all participating prior to inclusion. The study was conducted in accordance with the ethical principles set forth in the Declaration of Helsinki (1975, revised in 2013) and in accordance with Good Clinical Practice guidelines. All patients with T1DM and CD who met the inclusion criteria and did not meet any of the exclusion criteria were included in the study. For the control group, it was initially planned to recruit the first 100 age-matched eligible children. However, due to non-attendance for ultrasonography, failure to provide blood samples, or loss to follow-up, approximately 60 healthy controls were ultimately included in the analysis.

The study group was constituted according to the following inclusion criteria: children aged under 18 years presenting with negative thyroid autoantibodies [anti-thyroid peroxidase (TPOAb), anti-thyroglobulin (TgAb)], and exhibiting normal serum levels of thyroid-stimulating hormone (TSH) and thyroxine (free T4; FT4). The study excluded patients with systemic diseases other than T1DM and CD, chromosomal abnormalities such as Down syndrome, metabolic diseases, suspected thyroid malignancy, patients with prior neck radiotherapy, patients with immune system dysfunction, and patients with evidence of thyroiditis on ultrasound [Hypoechoic (low-reflection/dark) parenchyma, heterogeneous (irregular/patchy) appearance, nodular structures, increased blood flow (vascularization), and changes in gland size], despite normal thyroid function tests and autoantibodies. The children participating in the study were aged between 4 and 18 years old. The study included three groups of patients. The initial cohort comprised patients who had been diagnosed with T1DM for a period exceeding one year following the honeymoon period. The second group comprised patients who had been diagnosed with CD for a period exceeding one year, with confirmation of the diagnosis through histopathological examination. The diagnosis of celiac disease was established through the histopathological examination of a minimum of six biopsy specimens obtained from the duodenum bulbus and antrum, in conjunction with the identification of elevated tissue transglutaminase IgA levels [13]. The third group comprised healthy children who did not have any chronic or autoimmune disease. The control group comprised healthy volunteers with no history of autoimmune disease, normal tissue transglutaminase immunoglobulin A (IgA) levels, normal total IgA levels, negative thyroid autoantibody status, and normal thyroid function test results. Thyroid sonography was performed in the control group as part of the study. A total of 54 patients with CD, 71 with T1DM, and 60 healthy controls were included in the study.

During the first months after diagnosis, the underlying autoimmune process is highly dynamic. In type 1 diabetes, islet autoantibodies may still be appearing or disappearing, and metabolic control is often unstable, especially in the context of recent diabetic ketoacidosis and rapid metabolic changes [14]. In celiac disease, similarly, the early post-diagnosis period is characterized by active intestinal inflammation, malabsorption and subsequent nutritional and metabolic recovery, which can transiently influence thyroid function and vascularity rather than reflect chronic autoimmune thyroid involvement. Second, we wanted to minimize the potential confounding effects of this acute systemic and metabolic phase on thyroid elastography measurements [15]. By requiring a minimum disease duration of 1 year, we aimed to study patients in a more stable phase of their primary autoimmune disease, where the autoimmune background is established, but thyroid autoantibodies may still be negative.

Anthropometric measurements were obtained utilizing the ADE M320600-01 scale (GmbH & Co., Hamburg, Germany), with calculations based on Centers for Disease Control and Prevention (CDC) references via an online program (www.childmetrics.org). Blood samples were collected for biochemical and hormonal testing following an overnight fast. Serum levels of TSH, FT4, triiodothyronine (free T3, FT3), TPOAb, TgAb, and hemoglobin A1c (HbA1c) were quantified utilizing electrochemiluminescence immunoassays on Cobas 8000 and 6000 systems (Roche). Anti-endomysium antibodies were quantified utilizing Helmed devices and Aesku kits (AESKU.GROUP, Wendelsheim, Germany), whereas tissue transglutaminase IgA was evaluated employing Seac Alisei devices and Orgentec kits (ORGENTEC Diagnostika GmbH, Mainz, Germany).

**Figure 1:**
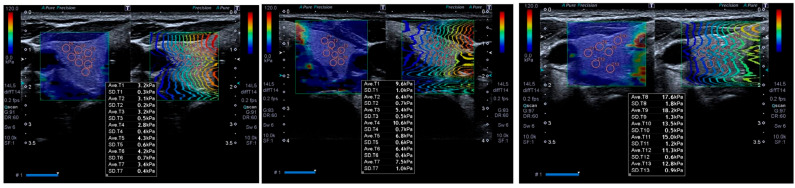
Images showing the calculation methods of the mean stiffness value (with standard deviations) of the thyroid gland using standard round ROIs in the subjects.

An experienced radiologist conducted thyroid ultrasonography and elastography using an Aplio 500 ultrasound system (Toshiba Medical Systems, Tokyo, Japan) with a 14 MHz linear probe. The total thyroid volume was calculated by measuring each lobe separately and excluding cases exhibiting evidence of thyroiditis. A region of interest measuring 2 mm in diameter was identified in the shear wave elastography (SWE) color map, which was recorded in kPa (Figure 1). At least 10 transverse measurements were taken from each lobe to determine the average thyroid stiffness.

Clinical and demographic data were extracted from hospital records using a standardized data collection form. All recorded variables were obtained at the time of study evaluation and verified for completeness. Data entry was performed by the research team and subsequently cross-checked to ensure accuracy. To maintain consistency, only measurements obtained during routine clinical follow-up under stable disease conditions were included. Cases with missing, incomplete, or inconsistent data were excluded from the final analysis. Anthropometric measurements and sonographic findings were collected prospectively for the purposes of this study and were not derived from routine hospital records.

### Statistical Analysis

The normality of the data was evaluated using the Shapiro–Wilk test, with variables exhibiting kurtosis and skewness within the range of −2 to +2 deemed to be normally distributed. Descriptive statistics were employed, comprising means, standard deviations, medians, and quartiles for numerical data, and counts and percentages for categorical data. Comparisons between two independent groups were conducted using either Student’s *t*-test or the Mann–Whitney U-test, while categorical variables were analyzed with the Chi-square test. A one-way analysis of variance (ANOVA) was employed for the comparison of independent groups, and the homogeneity of variances was evaluated through the utilization of Levene’s test. In instances where the homogeneity assumption was not met, Welch’s ANOVA was employed to analyze the variables. Conversely, the Kruskal–Wallis test was utilized for variables that exhibited non-normal distribution. Subsequent analyses were conducted using the Tukey multiple comparison test. A correlation analysis was conducted to evaluate the relationship between the variables. The Pearson correlation coefficient was employed for data that exhibited a normal distribution, whereas the Spearman correlation was utilized for data that did not. The statistical analysis was conducted using the IBM SPSS Statistics software, version 24.0. All analyses were conducted with a 95% confidence level, with *p* < 0.05 deemed to be statistically significant.

## 3. Results

**Figure 2:**
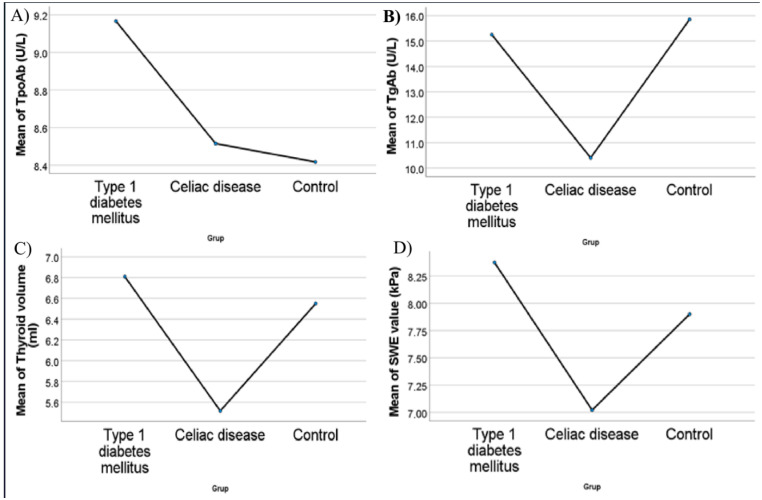
Box plots in case groups with (**A**) TPOAb, (**B**) TgAb, (**C**) Thyroid volume, (**D**) SWE value.

The study cohort comprised 185 children, of whom 71 (38.4%) had T1DM, 54 (29.2%) had CD, and 60 (32.4%) constituted the healthy control group. The participants ranged in age from four to 17.9 years, with a mean age of 11.4 ± 3.8 years. The gender distribution did not differ significantly between the groups. The children were categorized according to their body mass index (BMI), resulting in three distinct subgroups: normal, obese, and malnourished. BMI classification did not differ significantly between the groups (*p* = 0.052). TPOAb levels did not differ significantly between the groups (*p* = 0.459). Thyroid volume or standard deviation score did not differ significantly between the groups. Corresponding SWE values in the T1DM, CD and control groups were 7.7 (6.0–9.3), 5.9 (5.2–7.9) versus 7.1 (6.0–9.6), respectively (*p* = 0.002). The distribution of demographic data and clinical features among the groups is presented in Table 1. Şekil 2’de Plots of case groups with TPOAb, TgAb, Thyroid volume and SWE value are shown in Figure 2.

The duration of diabetes in the diabetic group exhibited considerable variation, ranging from 1 to 13 years, with an average of 3.6 ± 2.7 years. The mean daily insulin dosage was 0.9 ± 0.3 units per kg, with a range of 0.3–1.8 units. The mean HbA1c level was 9.5 ± 2.5%, with a range of 6% to 15.8%. In the cohort diagnosed with celiac disease, the time elapsed since diagnosis ranged from one month to 12 years, with an average of 1.7 ± 1.8 years. Serological analysis of celiac patients revealed tissue transglutaminase IgA levels of 47.5 ± 68.1 U/L and anti-endomysium antibody levels of 100.1 ± 126.2 U/L. The endoscopic biopsy results indicated that 4 cases were at stage 2 (9.3%), 10 were at stage 3A (18.5%), 29 were at stage 3B (53.7%), and 10 were at stage 3C (18.5%).

A summary of the correlation between thyroid SWE scores and clinical/laboratory parameters is provided in Table 2. SWE scores were significantly correlated with TgAb, thyroid volume, mean HbA1c, and time elapsed from a diagnosis of celiac disease.

## 4. Discussion

The objective of the study was to identify the high-risk groups for developing thyroiditis while being autoantibody negative in patients with T1DM and CD, which are diseases in which Hashimoto’s thyroiditis is frequently observed. Higher SWE values were significantly associated with T1DM. Additionally, SWE scores were significantly correlated with TgAb, thyroid volume, mean HbA1c, and time elapsed from a diagnosis of celiac disease. When the outcomes were evaluated, it was thought that especially CD patients had high SWE scores and that as the value increased, more stringent follow-up was necessary.

SWE can measure the stiffness of the thyroid gland in terms of velocity. Higher mean velocities were significantly associated with Hashimoto’s and Graves’ disease. However, mean velocity values did not differ significantly between Hashimoto’s and Graves’ disease. The optimal threshold mean velocity value was 2.19 m/s with 93% specificity and 95% positive predictive value [16]. The present study revealed that higher SWE values were significantly associated with T1DM. Two distinct explanations have been proposed to account for this controversial outcome. One potential explanation for this outcome is the higher probability of developing HT in T1DM patients compared to CD patients [17]. A second potential explanation for this finding may be related to the follow-up periods of chronic diseases in our study. The mean time since diagnosis for T1DM patients and CD patients was 3.6 ± 2.7 years and 1.7 ± 1.8 years, respectively. The existing literature identifies age at diagnosis and duration of gluten exposure as risk factors for the development of HT in CD cases [18,19]. SWE scores were significantly correlated with the time elapsed from a diagnosis of celiac disease. This indicates that a longer disease duration may be associated with increased inflammation. Furthermore, correlation did not differ significantly between tissue transglutaminase and anti-endomysium antibody levels and SWE values. Nevertheless, the HbA1C value and the SWE value were significantly correlated. The results of one study indicated that SWE values did not differ significantly between the healthy control group and the T1DM group [20]. It should be noted that this study differs from our own in that patients with autoantibodies were not excluded from the study.

A considerable body of research has demonstrated a positive correlation between TPOAb and TgAb and SWE values in patients with autoimmune thyroiditis [21,22]. Similarly, our study revealed a positive correlation between TgAb titers and SWE values. It is postulated that the lack of correlation between TPOAb and SWE scores is attributable to the fact that all patients exhibited autoantibody values within the normal range.

It has been demonstrated that SWE values in patients with HT are elevated in comparison to those observed in healthy subjects [23,24,25,26,27,28]. The SWE value of 12 was identified as the optimal cut-off point for the diagnosis of autoimmune thyroiditis, exhibiting 88.9% sensitivity, 93.7% specificity, 95.2% positive predictive value, and 85.5% negative predictive value [Area under curve (AUC) = 0.943] [25]. Different reference points can be used in strain elastography measurements. The predictability of autoimmune thyroiditis was significantly associated with the reference point, and using the trachea as a reference improves diagnostic performance [29]. In children who had recently been diagnosed with HT, SWE values were observed to be elevated relative to those of healthy children [24]. In children, SWE below 1.75 m/s suggests a lower likelihood of diffuse thyroid disease [30]. Given that the loss of elasticity due to fibrosis can occur even at the time of a new diagnosis, the objective of our study was to ascertain whether this loss occurs prior to the development of thyroiditis. The main outcome of our study was the identification of high SWE values as an independent risk factor for the onset of HT in children with CD and T1DM exhibiting negative autoantibody levels. Patients with elevated values can be monitored more frequently, while those with lower values can be observed at longer intervals.

In asymptomatic infants, thyroid volume increases with age, while firmness has been shown to remain constant for up to 180 days [31]. In addition, elastography is supported in studies as being helpful in identifying the malignancy potential in thyroid nodules and in selecting the correct location for fine needle aspiration biopsy [32,33,34,35,36]. Future prospective and longitudinal studies are needed to evaluate whether increased thyroid stiffness can predict the development of autoimmune thyroiditis in seronegative patients. Larger multicenter studies are also required to validate these findings and establish standardized pediatric reference values.

This study has some limitations. The first of these limitations is the retrospective design of this study. Problems such as bias and misremembering may arise due to this retrospective nature. The second limitation is the relatively small number of patients. A significant shortcoming is that elastography is not yet a standardized screening tool for autoimmune thyroiditis. Other limitations include insufficient family registries for polyglandular autoimmune syndrome, the need for long-term follow-up of autoimmune outcomes, and the study being conducted in a single center. Notwithstanding the aforementioned limitations, the implementation of an effective recording system and the fact that patients’ laboratory data are typically recorded within the system serve to mitigate these limitations.

## 5. Conclusions

This study employed non-invasive thyroid elastography in patients with T1DM, CD and control cases who exhibited negative thyroid autoantibody levels. The SWE scores were observed to be higher in children with T1DM compared to those with CD.

## Figures and Tables

**Table 1:** Clinical and laboratory characteristics of the groups.

	Type 1 Diabetes *n* = 71	Celiac Disease *n* = 54	Healthy Control *n* = 60	*p*
Gender				0.600 ^c^
- Female	33 (46.5%)	26 (48.1%)	33 (55.0%)	
- Male	38 (53.5%)	28 (51.9%)	27 (45.0%)	
Age (years)	11.9 ± 3.6	10.5 ± 4.1	11.7 ± 3.7	0.097 ^a^
Weight (kg)	41.3 ± 14.4	34.2 ± 18.2	46.8 ± 20.9	0.001 ^w^
Height (cm)	145 ± 17	135 ± 22	146 ± 19	0.004 ^w^
BMI (kg/m^2^)	17.9 (16.3–19.9)	16.7 (14.8–18.3)	19.3 (16.0–24.3)	<0.001 ^k^
Weight SDS	−0.1 ± 1.0	−0.6 ± 1.2	0.3 ± 1.2	<0.001 ^a^
Height SDS	−0.2 ± 1.0	−0.7 ± 1.3	−0.1 ± 1.0	0.002 ^a^
BMI SDS	0.1 ± 0.9	−0.2 ± 1.3	0.4 ± 1.1	0.014 ^w^
TSH (miU/mL)	2.1 ± 1.1	2.6 ± 1.2	3.0 ± 1.6	<0.001 ^w^
Free-T4 (ng/L)	13.3 ± 2.2	13.8 ± 1.8	13.2 ± 1.8	0.252 ^a^
Free-T3 (ng/dL)	3.5 ± 0.7	4.0 ± 0.6	4.0 ± 0.6	<0.001 ^a^
TPOAb (U/L)	8 (7–10)	8 (6–10)	8 (6–10)	0.459 ^k^
TgAb (U/L)	15 (12–17)	10 (10–11)	15 (12–17)	<0.001 ^k^
Thyroid volume (mL)	6.8 ± 2.9	5.5 ± 3.5	6.5 ± 3.2	0.056 ^a^
Thyroid volume SDS	1.2 ± 1.1	0.7 ± 1.3	0.9 ± 1.3	0.096 ^a^
SWE value (kPa)	7.7 (6.0–9.3)	5.9 (5.2–7.9)	7.1 (6.0–9.6)	0.002 ^k^

Anova ^a^ and Welch’s Anova ^w^ tests were performed in groups with normal distribution (Mean ± SD). Kruskal–Wallis test ^k^ was used for those who did not have a normal distribution [Median (Q1–Q3)]. Categorical data were tested using the chi-square ^c^ test. BMI: body mass index, SDS: standard deviation score, TSH: thyroid-stimulating hormone, TPOAb: anti-thyroid peroxidase, TgAb: anti-thyroglobulin, SWE: shear wave elastography, kPa: kilopascal.

**Table 2:** Relationship between thyroid shear wave elasticity (SWE) scores and clinical and laboratory parameters.

Parameter	R Coefficient	*p*
Age (years)	0.423	<0.001
Weight (kg)	0.455	<0.001
Height (cm)	0.364	<0.001
BMI (kg/m^2^)	0.403	<0.001 *
Weight SDS	0.257	<0.001
Height SDS	0.257	0.443
BMI SDS	0.150	0.041
TSH (miU/mL)	−0.034	0.650
Free-T4 (ng/L)	−0.084	0.253
Free-T3 (ng/dL)	−0.120	0.123
TPOAb (U/L)	0.131	0.076 *
TgAb (U/L)	0.157	0.033 *
Thyroid volume (mL)	0.447	<0.001
Thyroid volume SDS	0.278	<0.001
Diabetes duration (years)	0.002	0.987
Daily insulin dosage (unit/kg)	0.182	0.134
Mean HbA1c	0.322	0.006
Tissue transglutaminase Ab (IgA)	−0.005	0.974
Anti-Endomysium Ab (IgA)	0.006	0.968
Duration after celiac diagnosis (months)	0.375	0.006
Marsh Classification stage	0.006	0.969

The Pearson correlation coefficient was used to examine the relationship between variables. * symbol indicates Spearman correlation was performed. Abbreviations: BMI: body mass index, SDS: standard deviation score, TSH: thyroid-stimulating hormone, TPOAb: anti-thyroid peroxidase, TgAb: anti-thyroglobulin, Ab: antibody, IgA: immunoglobulin A.

## Data Availability

The data presented in this study are available on request from the corresponding author due to patient confidentiality and data protection regulations.
